# Active Ingredients of Voice Therapy for Muscle Tension Voice Disorders: A Retrospective Data Audit

**DOI:** 10.3390/jcm10184135

**Published:** 2021-09-14

**Authors:** Catherine Madill, Antonia Chacon, Evan Kirby, Daniel Novakovic, Duy Duong Nguyen

**Affiliations:** 1 Voice Research Laboratory, Discipline of Speech Pathology, Faculty of Medicine and Health, The University of Sydney, Sydney, NSW 2006, Australia; antonia.chacon@sydney.edu.au (A.C.); evan.kirby@sydney.edu.au (E.K.); daniel.novakovic@sydney.edu.au (D.N.); duong.nguyen@sydney.edu.au (D.D.N.); 2 Faculty of Medicine and Health, Central Clinical School, The University of Sydney, Sydney, NSW 2006, Australia

**Keywords:** Sob Voice Therapy, Optimal Phonation Task, Negative Practice, auditory-perceptual analysis, acoustic voice analysis

## Abstract

Background: Although voice therapy is the first line treatment for muscle-tension voice disorders (MTVD), no clinical research has investigated the role of specific active ingredients. This study aimed to evaluate the efficacy of active ingredients in the treatment of MTVD. A retrospective review of a clinical voice database was conducted on 68 MTVD patients who were treated using the optimal phonation task (OPT) and sob voice quality (SVQ), as well as two different processes: task variation and negative practice (NP). Mixed-model analysis was performed on auditory–perceptual and acoustic data from voice recordings at baseline and after each technique. Active ingredients were evaluated using effect sizes. Significant overall treatment effects were observed for the treatment program. Effect sizes ranged from 0.34 (post-NP) to 0.387 (post-SVQ) for overall severity ratings. Effect sizes ranged from 0.237 (post-SVQ) to 0.445 (post-NP) for a smoothed cepstral peak prominence measure. The treatment effects did not depend upon the MTVD type (primary or secondary), treating clinicians, nor the number of sessions and days between sessions. Implementation of individual techniques that promote improved voice quality and processes that support learning resulted in improved habitual voice quality. Both voice techniques and processes can be considered as active ingredients in voice therapy.

## 1. Introduction

A muscle-tension voice disorder (MTVD) is a commonly occurring dysphonia that results from disorganisation or dysfunction of the laryngeal musculature [1]. It can occur as a primary condition without organic changes to the vocal folds or as a secondary, compensatory condition to underlying organic or neurological laryngeal pathology. The aetiology of MTVD can be multifactorial and includes phonotrauma, excessive vocal load, glottic incompetence (vocal fold paresis and atrophy), psychological stress, and co-occurring medical conditions such as upper respiratory tract infection, laryngopharyngeal reflux, and sinusitis with post-nasal drip [2,3]. Within the voice-disordered population, functional dysphonia has documented prevalence rates of between 20.5 to 41%, while the prevalence of phonotraumatic lesions (e.g., vocal nodules and polyps) is 12–15% [4,5]. The majority of MTVDs are preventable [2] and early intervention is recommended to mitigate the negative impact of the disorder [6].

### 1.1. Behavioural Voice Therapy Is the First-Line Treatment for MTVD

Treatment of MTVD requires voice therapy as the first line of treatment [7], alongside the medical management of co-existing or contributing medical conditions. Both indirect and direct voice therapies are utilised in the treatment of MTVD in adults and children [8,9,10]. Indirect voice therapy, also termed vocal hygiene, aims to facilitate an individual’s vocal rehabilitation by identifying and eliminating poor vocal behaviours or other constraints to good vocal health, while promoting vocal health. Direct voice therapy describes a large range of individual vocal techniques and structured programs designed to change the habitual movement of the vocal system during phonation [8] such that the vocal needs of the individual are met without deterioration in the sound or sensation of phonation. Numerous systematic reviews and an increasing body of evidence have demonstrated that voice therapy is effective for the majority of patients with MTVD [11]; however, there is insufficient evidence to determine if one treatment is more effective than another. While some research has demonstrated that speech and language pathologists (SLPs) use a common approach to therapy [12], it is also well documented that SLPs use more than one MTVD therapy technique at a time [9,10]. This prevents clear identification of the therapeutic effect of each component of the treatment regime prescribed by the clinician. Therapies for MTVD are also very heterogenous and target different aspects of voice production. In addition, different therapies employ different conceptual approaches and there is a paucity of outcome data on the individual treatment components thought to modify voice production towards more optimal function.

There is a pressing need to ensure that the most cost-effective treatments are used, that is, treatments that provide evidence-based treatment effects with the maximum therapeutic effect in the minimum amount of time. Average treatment times for dysphonia across 140 research publications were documented as approximately consisting of 11 sessions of mostly 30 or 60-min durations, with average clinician-to-client face-to-face time estimated at 8.17 h [13]. The authors acknowledge that this was a conservative analysis, with many studies using fixed-treatment designs and others documenting clinical outcomes in North America, in which health insurance rules may influence intervention length and cost. If treatment efficacy can be improved, time and health-care costs may be reduced without compromising treatment outcomes, nor patient-centred care [14].

### 1.2. What Is an Active Ingredient in Voice Therapy?

The definition of an active ingredient has been recently considered in allied health and speech language pathology (SLP), specifically in [15,16,17]. Nevertheless, behaviours that generate a therapeutic effect can be difficult to identify in behavioural therapies due to a number of challenges. These include the lack of clarity surrounding rehabilitation ingredients, the fact that rehabilitation treatments often attempt to change multiple interacting patient functions, and a lack of standard nomenclature and definitions for specific treatment ingredients [18]. The treatment of voice disorders is one area in which significant efforts are being made to identify active ingredients in detail.

Quantifiable ingredients such as dosage, frequency, and intensity were initially proposed as active ingredients in SLP [16]. In recent times, a more expansive consideration of those components of a therapy that may have a therapeutic effect has been modelled in the Taxonomy of Voice Therapy [19]. This model proposes that treatment components may be classified into direct interventions (subdivided into auditory, somatosensory, musculoskeletal, respiratory, and vocal function), intervention delivery models (extrinsic and intrinsic), and indirect interventions (pedagogy and counselling) with more specific interventions listed under each sub-category [14]. The Rehabilitation Specification System (RTSS) [18] describes a simpler theoretical framework and proposed methodology by which treatments can be described according to a singular treatment target (the patient function that is to be changed by the ingredient(s)); one or more ingredients (what the clinician does to modify the target); and the mechanism(s) of action of the treatment [16]. Both the Taxonomy of Voice Therapy and the broader RTSS provide complex and detailed theoretical models that can inform our understanding; however, these models defining active ingredients are yet to be tested in clinic-based research.

Verdolini provides a simpler conceptualisation of the mechanisms of action as being divisible by the ‘what’ (the vocal technique) and the ‘how’ (the modality by which the change of function is learned) [20]. Across different voice therapies, the ‘what’ can vary from a single technique, such as Conversation Training Therapy (CTT) (use a clear voice) and Resonant Voice Therapy (RVT) (feel the buzz and notice the ease of phonation), to multiple technique therapies, such as Vocal Function Exercises (VFE) [21] (four distinct exercises targeting the whole vocal system), stretch and flow therapy [22], and the Accent Method [23,24]. The ‘how’ of learning to habituate the new vocal technique is remarkably homogenous across voice therapies [25] and involves processes originally described in motor learning research, such as task variation (hierarchical or end goal target) and negative practice.

There is little existing research on voice-disordered populations investigating the effectiveness of specific techniques and/or processes, as most research designs have evaluated the impact of the whole therapy rather than its component parts or stages. Most voice therapy programs that aim to provide a standardised series of voice exercises have been evaluated in controlled clinical trials [21,22,23,24,26,27]. All of these programs consist of multiple exercises that may be hierarchical in nature (e.g., Lessac Madsen Resonant Voice Therapy and RVT) or address different aspects of vocal function (e.g., VFE and the Accent Method). All have demonstrated efficacy with a range of effect sizes demonstrated across a variety of voice outcome measures; however, none have systematically evaluated the effect of each component or ‘ingredient’ in the treatment provided. Preliminary research investigating individual effects of components of VFE has isolated the therapeutic effects of practise dosage and the use of a semi-occluded vocal tract (nasal sound) [28,29]; however, this research was conducted in controlled experimental conditions with non-voice-disordered volunteers.

### 1.3. VoiceCraft^®^ Sob Voice Therapy

VoiceCraft^®^ Sob Voice Therapy (SVT) [30] is a direct voice therapy program whereby discrete individual techniques and processes are introduced at specific times and thus provides an opportunity to isolate possible effects of individual ingredients. Voicecraft^®^ is an SLP-directed voice therapy treatment model developed in the 1980s based on the work of numerous voice-science researchers and clinicians [31]. Described as a differentiated vocal tract model of vocal training that aims to develop the control of specific muscular movements in the larynx [32], it consists of a range of treatment programs for different patient populations (e.g., Yell Well for children with vocal nodules) that can be adjusted to the individual presentation of the patient depending on the type of voice condition, their individual muscular function in the larynx, and/or awareness of perceptual outcomes of phonation. This approach to the remediation of functional voice disorders has not been documented previously. Voicecraft training has proven to be effective in improving voice quality in healthy subjects [33] and to ‘fatigue proof’ the voice under conditions of sleep deprivation [33]. Despite being used across Australasia, Singapore, Europe, and the UK to treat voice and resonance disorders in adults and children, efficacy of Voicecraft^®^ programs, such as Sob Voice Therapy, has not been reported in voice-disordered populations.

Sob Voice Therapy is used to treat adolescent and adult patients with MTVD with or without organic change. The program consists of up to four techniques (as required) and utilises two common learning processes to support the generalisation and maintenance of the new voice techniques, namely task variation and negative practice (Table 1). It follows a hierarchical progression from an initial exercise utilising the most common features of voice therapy exercises, namely the optimal phonation task (OPT), followed by the introduction of sob voice quality (SVQ), the so-called heartbroken voice quality, and then habitual speech quality. Twang voice quality can be taught to assist in the production of loud voicing without effort, should the patient require this skill to meet their vocal needs. Task variance and negative practice are used in between the introduction of each technique. The difference between each technique can be physiologically and perceptually described according to the targeted activation of muscle groups that result in measurable movement outcomes. For example, the difference between OPT and SVQ involves targeting a lower larynx potion and some degree of laryngeal tilt in SVQ compared to OPT.

VoiceCraft^®^ and SVT describe voice therapy techniques that are based on a dynamical systems approach which acknowledges that the vocal system, like other complex movement systems, is self-organising [34]. Identifying the component of vocal function that is the most disorganised is the focus of the treatment and in the case of MTVD, it relates to some aspect of laryngeal function; for example, differentiated control of the adduction of the true vocal folds and retraction of the false vocal folds, and/or lowering of the larynx. Specifically, primary movements are targeted as these are implicated across a number of presenting symptoms (e.g., supraglottic constriction is associated with degraded voice quality and increased vocal effort). In this way, targeting a single movement, such as the widening of the supraglottic area via the release of laryngeal constriction manoeuvres, that then may address multiple aims, presents an efficient process of treatment, as multiple symptoms are addressed in one movement adjustment. Other aspects of the phonatory system such as breathing and resonance are de-emphasized unless they are the primary source of dysfunction, as it is presumed the neural system will automatically reorganise these functions around the biomechanical movement that is reorganised/optimised. For example, breathing is assumed to be mediated by communicative intent [35,36]. Different learning processes may have greater effect in the learning of the new, more optimal movement.

### 1.4. Retrospective Cohort Analysis vs. Randomised Control Trial

Given the value of voice therapy programs as the first line of treatment for commonly occurring MTVDs, understanding which treatment programs are effective and estimating their potential ‘active ingredients’ is essential. Despite being considered the highest level of evidence, the use of randomized controlled trials (RCTs) in investigating the treatment efficacy of voice therapies on voice disorders presents certain difficulties. Firstly, it is ethically challenging to allocate patients into different study arms given the need to recover the voice of professional voice users. Secondly, cost-effectiveness is a barrier to both clinicians and their patients, as most voice therapy programs require a course of weeks to months to complete. Lastly, patient compliance and the impact of various co-factors and comorbidities/medical conditions are amongst the burdens that can interfere with the intervention outcomes and how these are interpreted. A retrospective review of existing clinical databases had advantages of bringing evidence from ‘real-world’ scenarios to help clinicians and researchers determine (1) whether a particular therapy program is effective and, in standardised treatment programs, (2) to compare different therapy components with respect to their treatment efficacy.

The aims of the present study were to:

(1) evaluate the overall treatment effects of the Sob Voice Therapy program on MTVD with and without mucosal lesions of the vocal folds;
(2) investigate the effects of ingredients within the Sob Voice Therapy program on treatment outcomes for patients with MTVD; and
(3) identify any diagnostic or service delivery factors that influence the efficacy of a specific technique or process.

It was hypothesized that: (1) Sob Voice Therapy, which includes two vocal techniques (OPT and SVQ) and two training processes (SVQ variant and NP), would be effective in the treatment of MTVD; (2) processes (task variation and negative practice) rather than techniques (OPT and SVQ) would demonstrate statistically significant treatment effects; and (3) session number, treatment duration, and diagnostic and service delivery factors would have significant effects on treatment outcomes.

## 2. Materials and Methods

### 2.1. Study Design

This was a retrospective file audit of an existing private practice speech pathology clinical database. This study was approved by the Human Research Ethics Committee of the University of Sydney (protocol number: 2019/529).

### 2.2. Participants

#### 2.2.1. Selection Criteria

Participants were included if they had received a diagnosis of primary or secondary MTVD from an Ear, Nose and Throat specialist (ENT). ‘Primary’ referred to MTVD without visible vocal fold mucosal lesions and ‘secondary’ referred to MTVD with slight associated mucosal changes related to vocal trauma, such as pre-nodular and swelling lesions.

Inclusion criteria included: (1) over 18 years of age; (2) diagnosis of MTVD by an ENT report based on laryngoscopy; (3) had attended at least one voice assessment and one voice therapy session, enabling pre and post-acoustic data baseline recordings prior to and following both the teaching and practise of the OPT; (4) received only Sob Voice Therapy components as described above; and (5) reported to have done some practise of the therapy component (technique or process) as recommended by the clinician.

Exclusion criteria included: (1) under 18 years of age; (2) missing an ENT laryngoscopy report/diagnosis; (3) had undergone surgery of the larynx or surrounding structures (e.g., thyroid surgery) throughout their voice intervention period; (4) neurological voice and speech problems (e.g., dysarthria) or predominant mucosal lesions (e.g., cysts, polyps, and neoplasms); (5) types of functional dysphonia not related to vocal trauma, e.g., puberphonia, presbyphonia, and transgender voice; (6) missing voice recordings for more than one data point other than the initial and final session; (7) voice recordings with severely aperiodic signals (type 3 and type 4 signals) [37], precluding fundamental frequency-based measures; (8) received instruction in another voice therapy technique or process not described in Sob Voice Therapy; and (9) patients who could not detect any change in the sound or sensation of their voice production regardless of their success in achieving voice change during the OPT trial therapy task in the initial assessment, as this would suggest a possible undiagnosed neurosensory or cognitive impairment.

#### 2.2.2. Sample Size Calculation

The required number of patients for the retrospective review was estimated using an online sample calculation tool called GLIMMPSE [38], as this has been recommended for calculating samples for repeated-measures study designs [39]. Parameters used included: power = 90%; Geisser-greenhouse corrected test; Type I error rate α = 0.05; outcome measures = harmonics-to-noise ratio (HNR); number of measurements = 3 (baseline and two post-therapy assessments); predictor variables = type of muscle-tension voice disorders (primary and secondary); treatment effects = [MTD type x harmonics-to-noise ratio interaction]; mean scale factor = 2; and variability scale factor = 1. Regarding the mean values to put into the formula, we used baseline HNR values taken from baseline data in a randomized control clinical trial by Nguyen and Kenny [21], in which HNR pre-treatment of primary MTD was 18.6 decibels (dB). Considering there has been no similar study design in the literature, we assumed the first treatment and second treatment resulted in a 3.8 dB improvement in HNR for the primary MTD group as observed in the Nguyen and Kenny study [21]. Mean baseline HNR for secondary MTD was taken from Wenke et al. [40] in which baseline HNR was 16.6 dB as their study used participants with both primary MTD and MTD with lesions such as vocal nodules. We assumed the first and second treatments resulted in a 2.9 dB improvement in HNR for the secondary MTD group as observed in their standard treatment protocols [40]. Standard deviation (SD) of HNR for the formula was set at 4.5 dB according to the study of Wenke et al. [40]. The calculation resulted in a sample size of 74 (patients).

### 2.3. Voice Therapy Programs under Review: Sob Voice Therapy

Sob Voice Therapy was delivered to the patients by six different SLPs who had completed a 4-day workshop in VoiceCraft^®^ and SVT [30]. All were certified practicing speech pathologists with experience in treating patients with MTVD ranging from 1 to 15 years. Therapy was delivered in a face-to-face, one-on-one service delivery model across six different sites in an office setting. Patients were charged a fee for service in all cases. Eighteen out of sixty-eight participants were treated by more than one clinicians. Patients were taught the specific technique or process and required to perform the technique or task to 80% accuracy as judged by the clinician before moving onto the next phase. All sessions were documented as being 60 min long (according to the clinical hour of 50 min face-to-face time and 10 min of note taking/administration). Patients were recommended to undertake a specific amount of daily practise in each technique and/or process. Recommendations were based on motor learning principles of high frequency, distributed variable, and randomised and context-variable practise [41]. Typically, patients were recommended to practise once an hour for between 1 and 3 min, aiming for 10 practise sessions/day. As the therapy is based on hierarchical additive fractionation, patients were required to add practise in a new technique or process to that of their previous practise, which also allowed for task variation and randomisation. Individual specific practise data was not collected routinely from patients; however, all patients reported some level of practise. The number of sessions required to meet 80% correctness in the technique/process ranged from 1.3 to 2.4, with the number of days between each technique/process ranging from 27.8–37.5.

Extracted data was collected at five time points: (1) at the initial session (baseline) after which the OPT was taught in the same session; (2) at the subsequent session in which it was judged by the clinician whether the OPT had been acquired and the next technique (SVQ) was taught (OPT-SVQ); (3) at the subsequent session in which the clinician judged that SVQ had been acquired and sob variants were taught (SVQ-SVQ variants); (4) at the subsequent session in which the clinic judges whether the SVQ variants had been acquired and NP was taught (SVQ variants-NP); and (5) at the beginning of the session following the introduction of the NP process (NP post-NP). The number of sessions and days between each of the time points varied due to variation in clinic attendance and time taken to acquire each technique/process. The modal number of sessions between each technique/process was 1 and modal number of days was 14 (Table 2).

### 2.4. Data Extraction

#### 2.4.1. Demographic Characteristics

During the initial voice assessment, a thorough case history interview was conducted. This supplemented the referral and case history information collected by a comprehensive case history questionnaire [42] and the patient reported outcomes (PROMS) data collected prior to the assessment session including both the Voice Handicap Index-10 (VHI-10) [43] and Reflux Symptom Index (RSI) [44] as a standard (data not reported here). Data about age, gender, occupation, MTVD type (primary and secondary), vocal load, lifestyle, and history of comorbidities were extracted.

#### 2.4.2. Extraction of Voice Recordings

Patient data was extracted and de-identified by authors AC and EK to ensure the first author was blinded to the identification of patient data to remove any risk of bias. All patients included in this review had high-quality audio recordings of a comprehensive voice assessment undertaken at baseline including the reading of the Rainbow Passage [45], the Consensus Auditory Perceptual Evaluation–Voice (CAPE-V) phrases [46], and the prolonged vowel (/a/). All voice signals were captured using an AKG C520 cardioid ear-mounted microphone [47] placed at a constant distance of 6 cm, 45° off the mouth axis, and were analogue-to-digital converted using a professional external sound card (Roland Quadcapture [48]) at 44.1 kHz and 16-bit resolution. The signals were processed and saved to a laptop computer using the Audacity sound editing software [49] in *.wav format. Calibration of the sound level in the voice signals was not undertaken. In subsequent treatment sessions, audio recordings were made at the beginning of each session of the Rainbow Passage, CAPE-V phrases, and prolonged vowel/a/for a minimum of 3 s.

### 2.5. Auditory–Perceptual Outcome Measures

This retrospective review used four auditory–perceptual parameters for outcome measures, including overall severity of dysphonia, roughness, breathiness, and strain. These outcome measures were evaluated using auditory–perceptual analysis, which is considered the gold standard for clinical voice assessment [50].

#### 2.5.1. Listeners

Two certified practicing SLPs (2 and 3.5 years of experience in clinical voice assessment, respectively) and one ENT surgeon (19 years of experience in voice assessment) participated in the perceptual analyses. The raters reported normal hearing and vision at the time of the study.

#### 2.5.2. Stimuli

Voice samples were edited to include the middle three seconds of the second attempt of the sustained/a/vowel production, the third CAPE-V phrase (CAPEV3), and the Rainbow Passage (‘When the sunlight…… at the end of the rainbow’). These tasks were combined into a single file in Audacity. To avoid variabilities related to unequal sound pressure levels/hearing levels of the samples, all stimuli were normalized for loudness using the command ‘Loudness Normalization’ in the program to ensure that the perceived loudness of stimuli was 23 loudness units full-scale (LUFS). The intensity level of stimuli ranged from 70 to 72 dB as measured in Praat [51] using default intensity settings. Stimuli from 35 patients were randomly repeated for testing intra-rater reliability. In total, 285 samples were used.

#### 2.5.3. Procedure

Raters judged the level of the four voice dimensions, including overall severity, roughness, breathiness, and strain, using a 100-point visual analogue scale (VAS) based on the items described in the CAPE-V protocol [46] and embedded in an online auditory–perceptual rating tool called Bridge2practice, which is an education and research platform developed for audio–perceptual learning and practise of speech pathology students [52]. Judgments were made by moving a slider between 1 and 100, representing the minimum and maximum level of the quality being rated, respectively. Listeners were required to listen to the voice tasks as many times as they wished using a headphone and to make a judgment by changing the position of the slider on the VAS line mentioned above. All voice tasks were randomized. Responses were registered in the rating platform and exported to an Excel spreadsheet. The CAPE-V rating includes other perceptual rating features such as pitch, volume, and resonance, as well as additional features such as fry and diplophonia; however, features were not rated in this dataset.

#### 2.5.4. Reliability of Auditory–Perceptual Analyses

Reliability was assessed using SPSS 24.0 [53]. Intraclass correlation coefficients (ICC) [54] were used to determine the level of agreement between the first and second (repeated) ratings (intra-rater reliability) and across listeners (inter-rater reliability). ICC was calculated using a two-way mixed model, consistency type, and single measure analysis [ICC (3,1)]. To assess the level of correlation, ICC < 0.5 indicates poor correlation, 0.5–0.75 indicates moderate correlation, 0.75–0.9 indicates good correlation, and >0.9 indicates excellent correlation [55]. Table 3 shows good to excellent intra-rater reliability for most of the rated voice dimensions. Table 4 shows moderate to good inter-rater reliability for all rated voice dimensions.

### 2.6. Acoustic Outcome Measures

Voice samples were edited in Audacity to extract the middle three seconds (s) of the sustained/a/vowels, CAPEV3, and the second and third sentences of the Rainbow Passage (RP23). RP23 is a standard task in the analysis of dysphonia in speech and voice (ADSV) [56], which was used for the acoustic analysis in the present study. The use of RP23 would allow for cepstral measures to be comparable with the previous studies that used this task [57]. The quality of audio recordings for all samples was checked using the signal-to-noise ratio (SNR) using a Praat script called ‘Speech-to-noise ratio/voice-to-noise ratio v.01.01’ [58]. Only samples with a SNR ≥ 30 dB were used for the acoustic analyses [59].

#### 2.6.1. Harmonics-to-Noise Ratio (HNR)

HNR quantifies the level of noise in the voice signals and intensifies it in pathological voices [60]. It has been found that HNR is correlated with the perceptual assessment of hoarseness [60] and vocal clarity [61]. HNR has been an important and commonly used outcome measure of voice treatment [62,63]. Praat 6.1.40 [51] was used to measure HNR from the middle 3-s segments from three trials of vowel samples and the averaged result (in dB) was used for the statistical analysis.

#### 2.6.2. Fundamental Frequency (F0)

F0 remains one of the most important frequency-based measures that has been extensively used to reflect voice changes associated with different laryngeal configurations, e.g., vocal fold dimension [64] and vocal fold stiffness [65]. F0 was measured in Praat from CAPEV3 and the full Rainbow Passage. The standard deviation of F0 (F0SD), which represent vocal stability [66], was measured from the sustained vowel/a/. All voice data with severely aperiodic signals (signal types 3 and 4) [37] were excluded from the F0 and HNR measurements. F0 settings in Praat are presented in Appendix A.1.

#### 2.6.3. Cepstral Peak Prominence: Non-Smoothed (CPP) and Smoothed (CPPS)

A voice cepstrum is measured using a Fourier transform of the logarithm power spectrum [67]. A cepstral peak is identified within the dominant ‘rahmonic’ corresponding to the fundamental period from which the cepstral peak prominence (CPP) is calculated as the amplitude between the peak and the regression line directly below it [68]. A signal with a highly periodic waveform and a clear harmonic structure would have a higher cepstral peak than aperiodic signals [68]. CPP has been shown to have stronger weighted correlations with overall voice quality than any other acoustic measure [69]. It has also been considered a significant predictor of dysphonic severity [70].

The acoustic analysis program ADSV [56] was used to measure cepstral peak prominence (CPP) in dB for the vowel, CAPEV3, and RP23 vocal tasks. CPP settings in ADSV are presented in Appendix A.2. CPPS was measured in Praat using recommended settings [71,72], which are shown in Appendix A.3. Smoothing before calculating the cepstral peak can improve the accuracy of estimation [73]. In Praat, the smoothing of the cepstral measurement followed the procedures by Hillenbrand and Houde [73] using 20-ms (10-frame) time-smoothing windows and 1-ms (10-bin) quefrency smoothing [51]. The first step involves averaging cepstral values over time, while the second step involves cepstra being averaged across the quefrency [51]. Both CPP and CPPS were used to allow the data to be comparable to the other studies that used either of these measures. We also expected that CPPS was more sensitive than CPP in detecting treatment outcome due to its smoothing algorithm.

#### 2.6.4. Cepstral/Spectral Index of Dysphonia

The Cepstral/Spectral Index of Dysphonia (CSID) reflects overall voice quality [57,74] and has been shown to have high sensitivity and specificity [57] in discriminating pathological aspects from normal voice quality [75]. CSID data were obtained automatically in ADSV for the vowel and CAPEV3 task, and were manually calculated for RP23 samples based on CPP, low/high spectral ratio (LH), and low/high spectral ratio standard deviation (SDLH) values measured in ADSV using the following formula [57]:

CSID of Rainbow Passage = 154.59 − 10.39 × CPP − 1.08 × LH−3.71 × SDLH

#### 2.6.5. Vocal Intensity

Vocal intensity was measured in Praat from the/a/vowel, CAPEV3, and the whole Rainbow Passage. It was used to validate the cepstral measures as previous research has found CPP measures to be affected by vocal intensity: CPP would increase when vocal intensity was elevated [76].

#### 2.6.6. Reliability Analysis of Acoustic Measurements

Baseline acoustic data for 30 patients were reanalysed for two acoustic measures that involved the manual selection of the analysis samples (HNR of the vowel and F0 of CAPEV3). Results from the two analyses were compared using ICC statistics. The results showed that, for HNR, ICC values were 1 for both single measures and average measures (*p* < 0.001). For F0 of CAPEV3, ICC = 0.999 for single measures (*p* < 0.001) and ICC = 1 for average measures (*p* < 0.001). These results demonstrated excellent inter-rater reliability of the acoustic analysis. CPP, CPPS, and CSID measures were analysed using the entire edited vocal samples, which involved no manual selection of the waveform. Therefore, reliability analyses were deemed not necessary for those measures.

### 2.7. Statistical Analyses

Data were managed in Microsoft Excel [77] and analysed using IBM SPSS Statistics v.24.0 [53]. Descriptive statistics were used to describe cohort characteristics. Prior to the analyses, normal distribution of the data was examined using Kolmogorov–Smirnov tests [78]. For continuous variables, mean, standard deviation (SD), range, median, and the interquartile range were used. For categorical data, frequencies and percentages were used. Changes in outcome measures over the treatment period were analysed using a linear mixed model with patients representing random effects and time point (baseline and the four treatment technique points) representing fixed effects. Gender, diagnosis (MTVD primary vs. secondary), and treating clinicians also represented fixed effects. Interaction between time and the fixed factors was calculated to determine the impact of the factors on the treatment outcome. Significant fixed effects of time were further tested using pairwise comparison with the Sidak adjustment for *p* values. One-way repeated-measures analysis of variance (ANOVA) was used to examine the effects of each individual treatment ingredient on auditory–perceptual and acoustic outcome measures by comparing data between baseline and after each treatment. Effect size was calculated using partial Eta squared (η^2^) with the values of 0.01, 0.1, and 0.25 indicating small, medium, and large effects, respectively [79].

Pearson’s correlation coefficient (r) was used to calculate the correlation between the number of therapy sessions and treatment duration, as well as the treatment outcome in which r = 0.1, 0.3, and 0.5 indicated small, medium, and large effects, respectively [80]. Where there were multiple calculations, the Bonferroni adjustment was applied to the *p* value. In all statistical analyses, a significance of *p* < 0.05 was used.

## 3. Results

### 3.1. Characteristics of the Study Population

In total, 68 participants were included in this study. Of these, there were 60 females (88.7%) with a mean age of 34.5 years (SD = 13.0, range = 20–84). There were eight males (11.3%) with mean age of 43.6 years (SD = 16.3, range = 25–70). In brief, 11 were vocal performers (16.2%), 49 were professional voice users (72.1%), and 8 belonged to other occupations (11.8%). Twenty-six had a history of vocal training (38.2%), 36 had not had voice training before (52.9%), and 6 did not provide information about voice training history (8.8%). Laryngeal assessment via ENT was reported to have been conducted on all 68 patients, which showed that 34 had primary MTD and 34 had MTD with mucosal lesions. The mean duration of voice problems was 19.2 months (SD = 26.5; 95% CI for mean = 12.5–25.9; minimum = 1.0; maximum = 132.0; median = 12.0; interquartile range = 18.0). The mean VHI-10 score was 17.8 (SD = 9.4; 95% CI = 15.5–20.1; minimum = 1; maximum = 38; median = 18.0; and interquartile range = 14.0). The study cohort was therefore considered typical of previously documented treatment-seeking populations with voice disorders reported in other studies [81,82]. Data on vocal load, history of comorbidities, and lifestyle are presented in Table A1, Table A2 and Table A3 in Appendix B.

**Figure 1:**
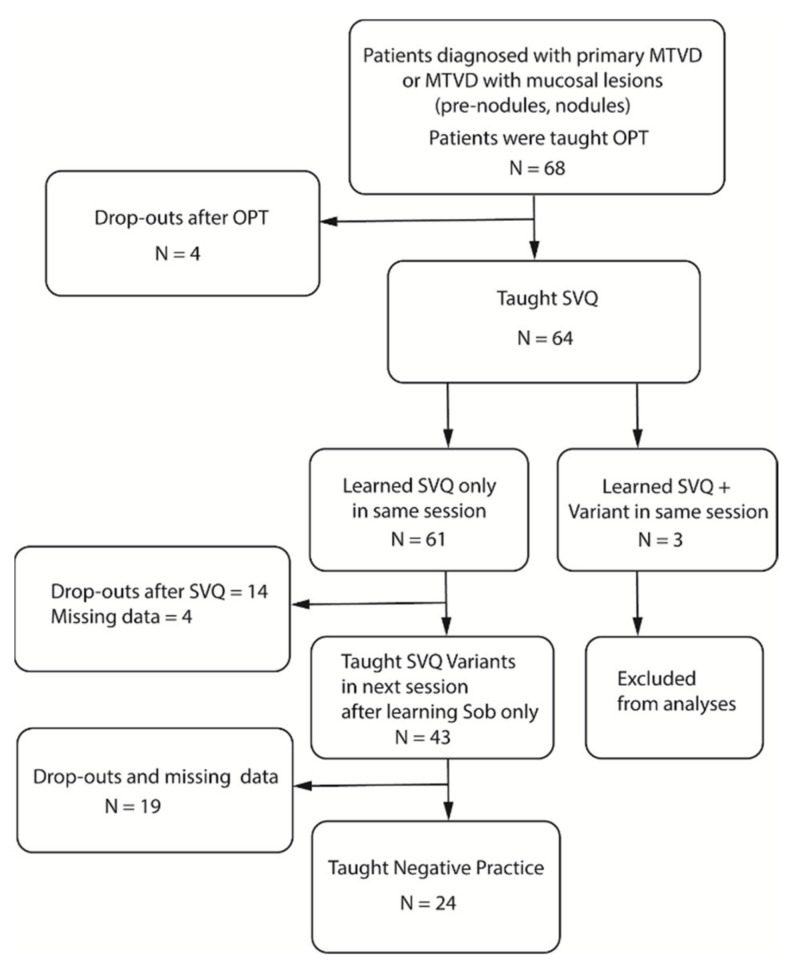
Flowchart of the treatment techniques.

Figure 1 shows the number of patients who underwent all four components of Sob Voice Therapy. For all participants (*n* = 68), the OPT was taught as the initial therapy exercise/laryngeal posture. Sixty-four participants (94.1%) went on to be taught SVQ as their second voice therapy exercise. Three (4.7%) were taught SVQ in addition to a SVQ variant (i.e., sob phrases or sob sirens) simultaneously in their second appointment. Of the 61 patients who were taught the OPT followed by SVQ, 43 (70.5%) were then taught SVQ variants, with most of these participants (*n* = 33) first being taught SVQ phrases. Fourteen out of sixty-one (22.9%) did not attend any further sessions following the successive teaching of the OPT and SVQ. Following teaching of the OPT, SVQ, and SVQ variants, 55.8% (*n* = 24/43) of participants were then taught the generalisation technique of negative practice, with the remaining 19 participants being lost to follow up or having incomplete data sets.

### 3.2. Treatment Effects of Sob Voice Therapy on MTVD

#### 3.2.1. Auditory-Perceptual Outcomes

The changes in perceptual outcome measures over time were calculated using a linear mixed model. Patients were treated as random effects and treatment (i.e., baseline and the four technique points) as fixed effects. Diagnosis (primary MTD and secondary MTD) was also a fixed factor to examine the interaction with treatment. The estimate of the fixed effects was based on the regression coefficient (b) for each effect associated with its 95% CI and the *p* value. Changes of the outcome measures over time were evaluated using multiple pairwise testing in which the Sidak adjustment for *p* values was applied.

• Overall severity ratings

**Figure 2:**
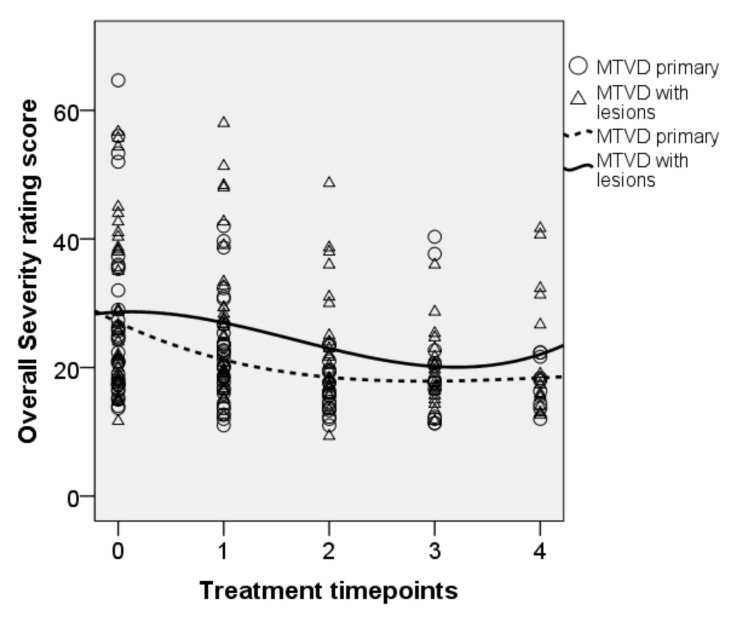
Longitudinal plot of data for the overall severity ratings. Trend line is shown for each subgroup. 0 = baseline, 1 = OPT, 2 = SVQ, 3 = SVQ variant, and 4 = NP.

Figure 2 shows rating scores of the overall severity of dysphonia for all time points. The overall progression, as indicated by the trend line, was that the rating scores were lower towards the final technique point (NP) for both diagnostic groups. There were significant fixed effects of treatment [F(4, 170.706) = 12.142, *p* < 0.001]. There was no significant effect of diagnosis (*p* = 0.125) and no significant interaction between treatment and diagnosis (*p* = 0.431). Parameter estimates showed a significant decrease in the overall severity ratings at the final technique point (NP) compared to baseline (b = 5.603, t = 3.047, *p* = 0.003). Compared with baseline, the mean (95% CI, Sidak-adjusted *p*) of the overall severity rating score decreased by 3.2 (0.4–5.9, *p* = 0.013), 6.9 (3.7–10.2, *p* < 0.001), 5.4 (1.7–9.0, *p* < 0.001), and 7.2 (3.0–11.4, *p* < 0.001) after treatments with OPT, SVQ, the SVQ variants, and NP, respectively.

• Roughness ratings

**Figure 3:**
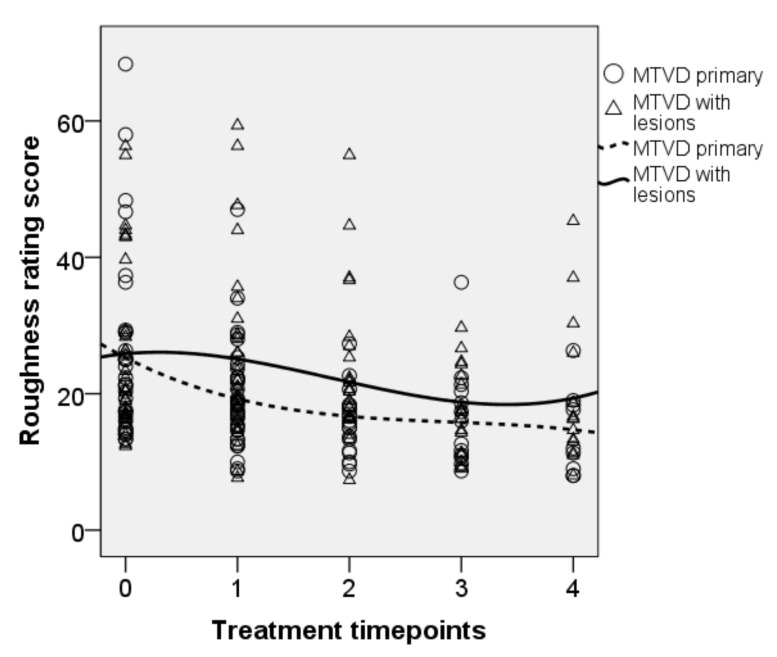
Longitudinal plot of data for the roughness ratings. Trend line is shown for each subgroup. 0 = baseline, 1 = OPT, 2 = SVQ, 3 = SVQ variant, and 4 = NP.

Figure 3 shows the changes of roughness rating scores over time with a steady decrease towards the end of the treatment program. The effects of the fixed factor ‘treatment’ on this outcome measure were significant [F(4, 171.467) = 10.082, *p* < 0.001]. The effect of diagnosis (*p* = 0.090) and interaction effects between treatment and diagnosis (*p* = 0.231) were not significant. Parameter estimates showed a significant decrease in the rating score of roughness after NP as compared to baseline (b = 4.842, t = 2.493, *p* = 0.014). The mean (95% CI, Sidak-adjusted *p*) of the roughness rating scores decreased by 3.5 (0.6–6.4, *p* = 0.007), 5.7 (2.3–9.2, *p* < 0.001), 6.4 (2.5–10.2, *p* < 0.001), and 7.3 (2.9–11.7, *p* < 0.001) after OPT, SVQ, the SVQ variants, and NP, respectively.

• Breathiness ratings

**Figure 4:**
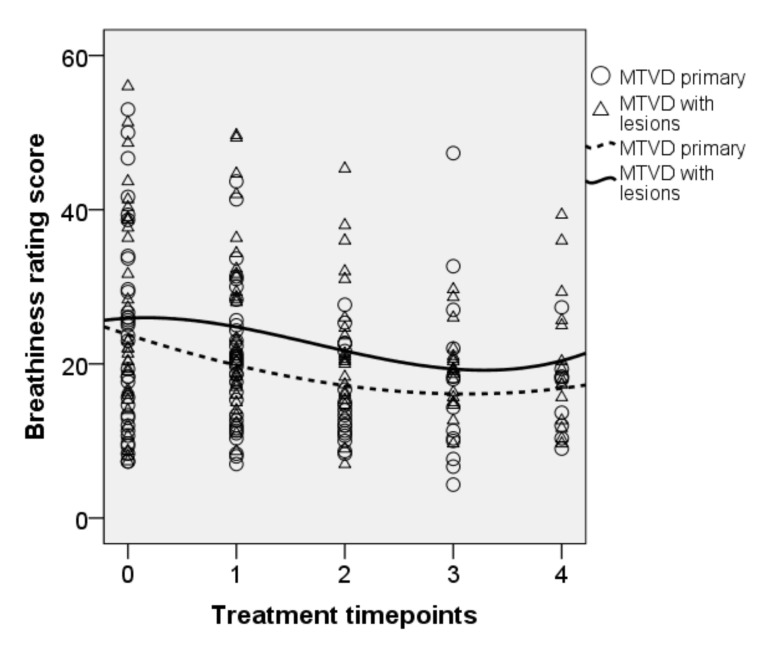
Longitudinal plot of data for the breathiness ratings. Trend line is shown for each subgroup. 0 = baseline, 1 = OPT, 2 = SVQ, 3 = SVQ variant, and 4 = NP.

Changes in the breathiness rating scores over the treatment period are presented in Figure 4, which shows a similar trend of decrease across the treatment techniques. There were significant effects of treatment [F(4, 170.294) = 5.482, *p* < 0.001], no significant effect of diagnosis (*p* = 0.102), and no significant interaction between treatment and diagnosis (*p* = 0.715). The decrease in breathiness rating scores after NP was significant as compared with baseline (b = 4.27, t = 2.13, *p* = 0.035). The mean (95% CI, Sidak-adjust *p*) ratings of breathiness decreased by 2.1 (−0.9–5.1, *p* = 0.367), 4.7 (1.2–8.2, *p* = 0.002), 4.1 (0.1–8.0, *p* = 0.040), and 5.8 (1.3–10.3, *p* = 0.004) after OPT, SVQ, the SVQ variants, and NP, respectively.

• Strain ratings

**Figure 5:**
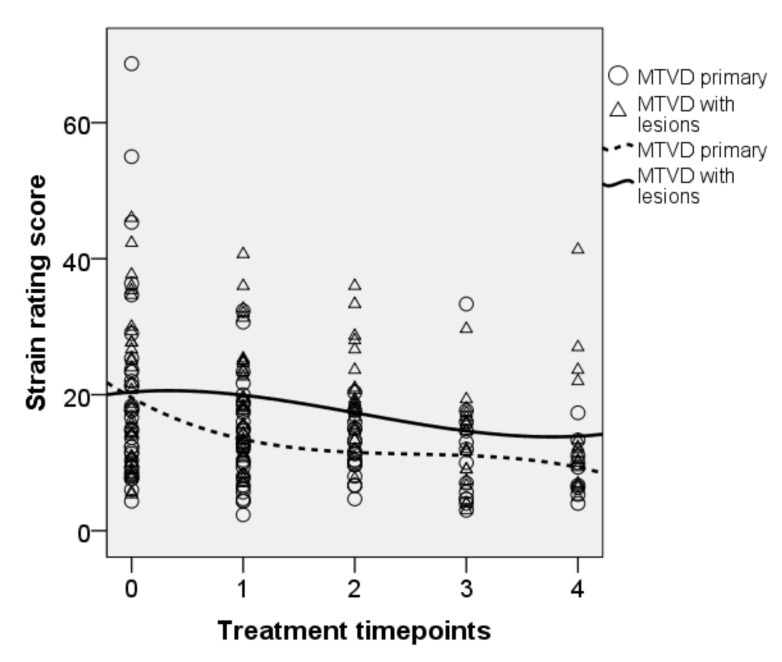
Longitudinal plot of data for the strain ratings. Trend line is shown for each subgroup. 0 = baseline, 1 = OPT, 2 = SVQ, 3 = SVQ variant, and 4 = NP.

Figure 5 shows the changes in the rating scores for strain quality after each technique. Overall, rating scores of this voice dimension decreased over the technique points. The trajectory of the trend lines shows that the rating scores for primary MTD decreased immediately at OPT while the decrease was not so obvious for MTD with lesions. There were significant effects of the fixed factors ‘treatment’ [F(4, 171.739) = 9.743, *p* < 0.001] and ‘diagnosis’ [F(1, 73.367) = 5.033, *p* = 0.028], and marginally significant interaction between treatment and diagnosis [F(4, 171.739) = 2.422, *p* = 0.05]. There was a significant improvement in this voice quality after the last time point (NP) as compared to baseline (b = 5.01, t = 2.643, *p* = 0.009). There were decreases in the mean (95% CI, Sidak-adjusted *p*) of 3.8 (0.9–6.6, *p* = 0.002), 3.7 (0.3–7.0, *p* = 0.021), 6.6 (2.9–10.4 *p* < 0.001), and 7.4 (3.1–11.7, *p* < 0.001) after OPT, SVQ, the SVQ variants, and NP, respectively.

#### 3.2.2. Acoustic Outcomes

• Harmonics-to-noise Ratio

**Figure 6:**
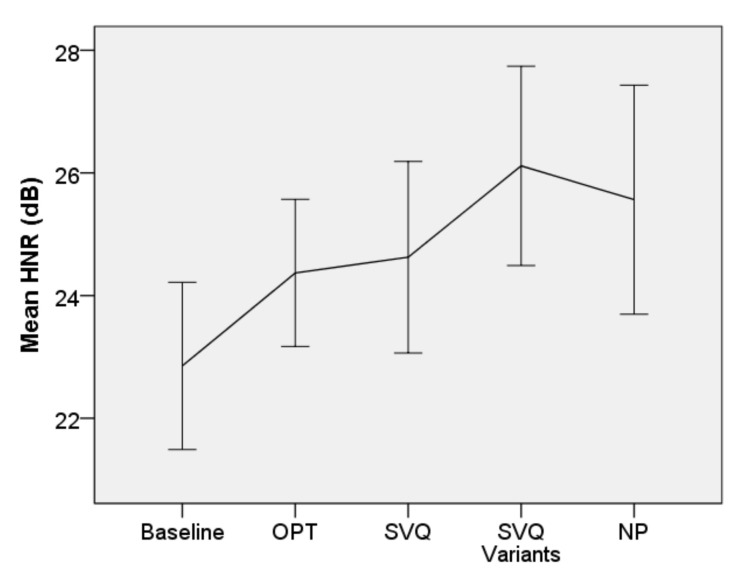
Mean harmonics-to-noise ratio. Error bars indicate 95% CI for the mean.

Figure 6 shows the mean HNR (dB) at baseline and at all the treatment time points. Significant effects of the treatment were found [F(4, 168.921) = 3.672, *p* = 0.007], while no significant interaction between treatment and diagnosis was present (*p* = 0.327), meaning that the effects of the treatment did not depend upon MTD type (primary or with mucosal lesions). The improvement in HNR between baseline and NP was significant (b = −2.82, t = −2.470, *p* = 0.014). The mean (95% CI, Sidak-adjusted *p*) of HNR (dB) increased by 1.6 (−0.2–3.3, *p* = 0.099), 1.7 (−0.3–3.7, *p* = 0.141), 2.5 (0.2–4.8, *p* = 0.022), and 2.4 (−0.3–5.2, *p* = 0.11) after OPT, SVQ, the SVQ variants, and NP, respectively.

• Fundamental frequency

Table 5 presents F0 data at baseline for all three vocal tasks. For F0 of CAPEV3, there were no significant fixed effects of treatment (*p* = 0.585) and no significant interaction between treatment and diagnosis (*p* = 0.358). There were also no significant effects of treatment (*p* = 0.276) and no significant interaction between treatment and diagnosis (*p* = 0.523) for the F0 of the Rainbow Passage.

F0SD (vowel) also showed significant effects of treatment (*p* = 0.716) and no significant interaction between treatment and diagnosis (*p* = 0.111).

• CPP

**Figure 7:**
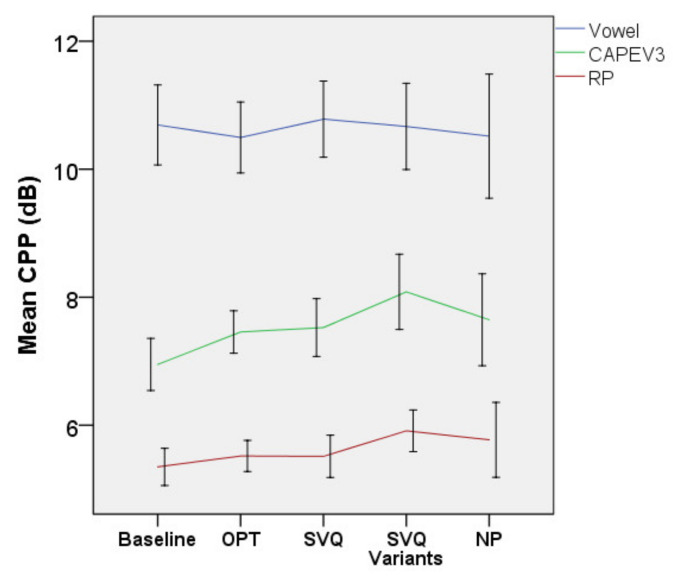
Cepstral peak prominence (CPP) of all vocal tasks. Error bars indicate 95% CI for the mean. Abbreviations: CAPEV3, third CAPEV phrase and RP, Rainbow Passage.

Figure 7 shows CPP data for all three vocal tasks. A significant effect of treatment was found for the CPP of CAPEV3 [F(4, 168.369) = 4.721, *p* = 0.001] but there was no interaction between treatment and diagnosis (*p* = 0.737). The improvement of CPP at NP as compared to baseline was significant (b = −0.915, t = −2.726, *p* = 0.007). Compared to baseline, the CPP of CAPEV3 only improved by 0.5dB after OPT (95% CI = −0.04–0.96, *p* = 0.088). After SVQ, the SVQ variants, and NP, the mean (95% CI, Sidak-adjusted *p*) of this measure (in dB) increased by 0.62 (0.04–1.21, *p* = 0.03), 0.8 (0.2–1.5, *p* = 0.006), and 0.8 (0.05–1.5, *p* = 0.03), respectively.

There was marginal fixed effect of treatment on the CPP of the Rainbow Passage [F(4, 171.130) = 2.312, *p* = 0.06]. Significant improvement in this measure was found after NP as compared to baseline (b = −0.442, t = −2.137, *p* = 0.034). Pairwise comparisons with baseline showed that the mean (95% CI, Sidak-adjusted *p*) of the CPP (dB) of the Rainbow Passage increased by 0.14 (−0.17–0.45, *p* = 0.912), 0.17 (−0.2–0.54, *p* = 0.879), 0.31 (−0.1–0.72, *p* = 0.284), and 0.45 (−0.02–0.92, *p* = 0.069) after OPT, SVQ, the SVQ variants, and NP, respectively.

There was no significant fixed effect of treatment (*p* = 0.849) and no significant interaction between treatment and diagnosis (*p* = 0.227) on the CPP of the vowel.

• CPPS

**Figure 8:**
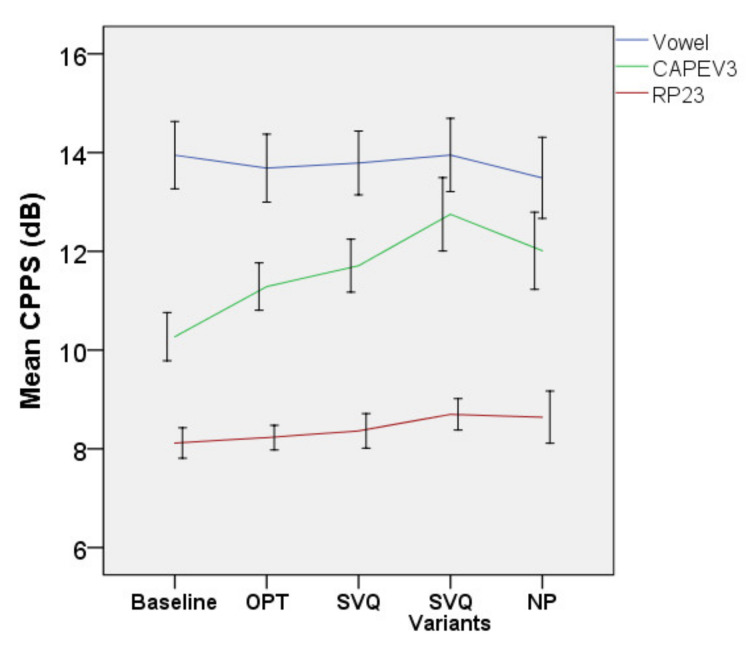
Smoothed cepstral peak prominence of all vocal tasks. Error bars indicate 95% CI for the mean. Abbreviations: CAPEV3, third CAPEV phrase and RP, Rainbow Passage.

Figure 8 shows CPPS data for all treatment time points. The CPPS of CAPEV3 demonstrated a steady increase from OPT towards the final technique (NP). There was a significant effect of treatment on this measure [F(4, 171.649) = 14.921, *p* < 0.001] but there was no significant interaction between treatment and diagnosis (*p* = 0.673), i.e., the changes in this measures over time were similar between primary MTD and MTD with lesions. The increase in the CPPS of CAPEV3 at NP as compared to baseline was significant (b = −1.985, t = −4.286, *p* < 0.001). The mean (95% CI, Sidak-adjusted *p*) of this measure (in dB) increased by 1.02 (0.33–1.7, *p* < 0.001), 1.43 (0.63–2.23, *p* < 0.001), 2.06 (1.17–2.95, *p* < 0.001), and 1.91 (0.88–2.94, *p* < 0.001) after OPT, SVQ, the SVQ variants, and NP, respectively.

The CPPS of vowels and RP23 are also shown in Figure 8. No significant effects of treatment were found for the CPPS of the vowel (*p* = 0.819) and RP23 (*p* = 0.156).

• CSID

**Figure 9:**
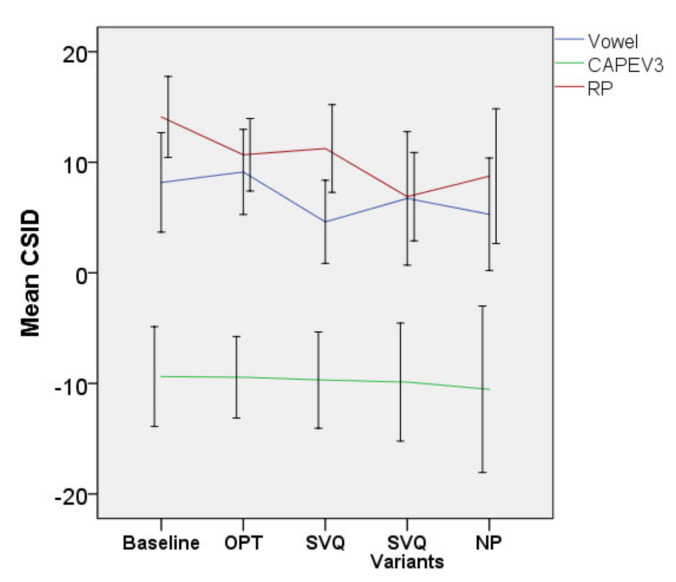
Mean CSID of all vocal tasks. Error bars indicate 95% CI for the mean. Abbreviations: CAPEV3, third CAPEV phrase and RP, Rainbow Passage.

Figure 9 shows the CSID of all tasks. There was a significant effect of treatment on the CSID of the Rainbow Passage [F(4, 170.887) = 2.859, *p* = 0.025]. No significant interaction effect between treatment and diagnosis was found (*p* = 0.161). The model showed a significant decrease in CSID after NP as compared with baseline (b = 6.04, t = 2.327, *p* = 0.021). Pairwise comparisons across time points showed that the mean (95% CI, Sidak-adjusted *p*) of CSID decreased by 2.82 (−1.07–6.7, *p* = 0.344), 2.51 (−2.11–7.13, *p* = 0.736), 4.33 (−0.8–9.47, *p* = 0.164), and 6.03 (0.16–11.89, *p* = 0.04) after OPT, SVQ, the SVQ variants, and NP, respectively.

The effects of the treatment for the CSID of the vowel (*p* = 0.683) and CAPEV3 (*p* = 0.935) were not statistically significant (*p* > 0.05).

• Vocal intensity

There were no significant fixed effects of treatment on the intensity of the vowel (*p* = 0.557), CAPEV3 (*p* = 0.357), and Rainbow Passage (*p* = 0.777).

### 3.3. Estimates of Active Ingredients within the Sob Voice Therapy Program

Apart from evaluating the treatment outcome of the whole Sob Voice Therapy program, we were also interested in estimating the effects of each of the individual therapy components (OPT, SVQ, the SVQ variants, and NP). This was evaluated via effect sizes, which were calculated as the Eta squared (η^2^) using one-way repeated-measures ANOVA for the differences in the outcome measures between baseline and after each technique point. This calculation was performed for auditory–perceptual and acoustic measures with statistically significant fixed effects of treatment. The data set for this calculation was *n* = 24 patients who had completed voice recordings at all mentioned time points. Patients with any missing data points were excluded from this analysis.

#### 3.3.1. Effect Size for Auditory–Perceptual Outcomes

Table 6 shows the mean (SD) and mean differences between baseline and each of the voice therapy techniques for all auditory–perceptual parameters. This table also presents the effect sizes corresponding to the results for the repeated-measures ANOVA. Overall, findings for auditory–perceptual ratings of overall severity, roughness, and breathiness showed that SVQ, the SVQ variants, and NP were active ingredients with large effect sizes. OPT did not demonstrate therapeutic effects. For strain ratings, only the SVQ variants and NP were the active ingredients.

#### 3.3.2. Effect Size for Acoustic Outcomes

Table 7 shows effect sizes associated with the outputs of the repeated-measures ANOVA for the changes in acoustic measures after each voice therapy ingredient as compared with baseline. Findings on the CPPS of CAPEV3 showed that SVQ, the SVQ variants, and NP were the active ingredients, and the last two ingredients (SVQ variant and NP) were associated with large effect sizes. Data of the CPP of CAPEV3 and CSID of the Rainbow Passage suggested that NP was an active ingredient.

Other acoustic measures did not show significant changes after the treatment techniques as compared with baseline. The effect sizes for acoustic measures with non-significant fixed effects of treatment are shown in Table A4 in Appendix C.

### 3.4. Impact of Service Delivery Factors on the Treatment Outcome

#### 3.4.1. Number of Sessions and Duration of Sob Voice Therapy

Bivariate correlation coefficients were calculated to examine the relationship between the treatment dose and the differences in the outcome measure values for each technique. For example, for OPT, the differences between baseline and post-OPT data were calculated, which were then used to calculate the correlation with the number of sessions and treatment duration. For OPT, there was no significant correlation between the number of therapy sessions, duration of voice therapy (weeks), and any of the pre/post differences in the auditory–perceptual and acoustic measures (*p* > 0.05). For SVQ and SVQ variants, there was no significant correlation between the number of sessions, duration of voice therapy, and the pre/post differences in the auditory–perceptual and acoustic outcome measures (*p* > 0.05). For NP, there were correlations between the number of sessions and the pre/post differences in both the roughness ratings (r = −0.49, *p* = 0.024) and strain ratings (r = −0.49, *p* = 0.024). After Bonferroni‘s adjustment for multiple correlation calculations, a significant *p* value would be 0.0035. Therefore, these were deemed not statistically significant.

#### 3.4.2. Clinician Effects

Due to the involvement of six SLPs in the treatment process across patients, the effects of the treating clinicians were examined using a factorial two-way ANOVA test [clinician × treatment] with repeated measures on ‘treatment’ (baseline, OPT, SVQ, SVQ variants, and NP). Main effects were calculated for the ‘clinician × treatment’ interaction. The results showed that there were no significant interaction effects between clinicians and the treatment for all perceptual and acoustic variables (*p* > 0.05). This suggested that all clinicians contributed the same amount of variance in the treatment outcome over time.

### 3.5. Drop-Out Rate

Ten out of 68 (14.7%) did not attend further therapy following their second appointment. Twelve participants (17.6%) did not attend further sessions after their third appointment. Eleven participants (16.2%) did not return to therapy following their fourth session.

## 4. Discussion

Voice therapy is a major therapeutic intervention that can be delivered as a stand-alone treatment or in combination with medical and/or surgical treatment. Early and effective voice therapy outcomes can prevent more complicated pathologies within the larynx that require costly treatment regimes. The purpose of this study was to retrospectively review clinical data from an SLP voice database to investigate the clinical outcomes of four components of a standardised voice therapy program (Sob Voice Therapy) and to provide preliminary data on the effects of its ‘active ingredients’. Statistical analyses involved the use of a linear mixed model, which allowed for the robust estimation of the treatment effects, given that patients were treated as random effects [88]. Patient factors such as history of comorbidities, voice use, and previous training were therefore considered random and were not specifically analysed. Treatment outcomes were evaluated using CAPE-V auditory–perceptual analysis, which is the "gold standard" of voice evaluation, and acoustic analysis including spectral-based measures (CPP/CPPS and CSID), which is an objective, non-invasive, and reliable evaluation with great sensitivity and specificity to voice changes [57,69,89]. These were believed to accurately reflect the treatment effects of the Sob Voice Therapy. Treatment sessions and timeframes were comparable to averages reported in the literature [13].

### 4.1. Treatment Effects of Sob Voice Therapy on Patients with MTVD

The first aim in the present study was to evaluate the treatment effects of SVT on MTVD. The study population consisted of typical treatment-seeking patients with primary MTVD (without obvious vocal fold mucosal lesions) or secondary MTVD (with mild mucosal changes deemed related to vocal hyperfunction, such as pre-nodules swellings and mucosal thickening) as these are the most common voice disorder types, representing approximately 40% of the case load in voice clinics [90]. The findings showed significant treatment effects in all auditory–perceptual measures for the whole treatment when compared to pre-treatment levels. There was a significant positive effect of SVT as measured by the decreased auditory–perceptual ratings of overall severity, roughness, breathiness, and strain between baseline and NP. Significant effects of treatment were also observed for acoustic measures such as HNR (vowel), CPP (CAPEV3 and Rainbow Passage), CPPS (CAPEV3), and CSID (Rainbow Passage). Notably, the HNR (vowel) value post-treatment is likely to have been judged perceptually clear compared to being not clear prior to treatment, based on [61]. However, no significant changes were found for F0, F0SD, and intensity (*p* > 0.05). These suggested that this voice therapy program was more effective in improving voice quality than in modifying pitch and loudness. The non-significant effects on F0SD also stemmed from the findings that the values of this measure were within normal ranges for both genders (Table 5).

For both auditory–perceptual and acoustic measures, the treatment effects did not depend upon the MTVD type, whether being primary or secondary. The significant effects of diagnosis observed for the auditory–perceptual ratings of breathiness and strain accurately reflected the MTVD type, with primary MTVD showing lower rating scores than secondary MTVD. This is expected with persistent associated laryngeal pathology that may affect voice quality.

Baseline values across outcome measures were indicative of predominantly mild MTVD in the cohort. For example, the mean auditory–perceptual rating score ranged from 18.7 for strain to 26.8 for overall severity (Table 6). Mean acoustic measure values were only marginally below cut-off values for voice disorder for CPP, while CSID values at baseline were within normative ranges (Table 7). The effects of the SVT on patients with more severe MTVD and on patients with predominantly mucosal lesions remain unclear and would need future studies to investigate if the same therapy components are ‘active ingredients’ in this cohort; signal typing as an outcome measure would be recommended in that case. Home practise dosage and frequency data was not collected, which precluded the analysis of home practise as an active ingredient. This study also lacked long-term follow-up, which impacts on the inference of the maintenance/sustainability of the outcome for this voice disorder. This study did not directly measure specific muscle-tension parameters or provide patient-reported outcome measures as outcome data, and not all participants were diagnosed by examination using videostrobolaryngoscopy. Prospective designs would address these issues.

### 4.2. Active Ingredients of the Sob Voice Therapy Program

Each technique within the SVT has a specific role. In OPT and SVQ, patients practised different techniques that targeted at a clear and effortless voice. In the SVQ variants and NP, patients practise specific exercises for generalising a clear and effortless voice to connected speech with intonation variation. We hypothesised that treatment effects in habitual voice quality would be observed after the SVQ variants and NP were introduced, that is, after the patient had practised exercises designed to facilitate generalisation of improved vocal function to habitual, connected speech contexts. The findings revealed that the SVQ, SVQ variants, and NP were the most active ingredients with small to medium effect sizes across the auditory–perceptual and acoustic measures of voice quality.

#### 4.2.1. Effects of OPT

As hypothesized, the findings showed that OPT was not a statistically significantly active ingredient to change voice quality in the habitual phonation of the cohort, despite resulting in improved voice outcome measures after this component was introduced. Auditory–perceptual outcome measures (Table 6) and acoustic measures, except the CSID of CAPEV3 (Table 7), demonstrated that the effects of OPT were not significant. The data on OPT may be explained by a range of factors. Firstly, the task is taught at the end of the initial assessment session with the purpose of raising perceptual awareness to the auditory–perceptual and kinaesthetic features of the voice, as well as providing cues to prime improved laryngeal function. The sound produced, however, is brief (less than 2 s as modelled) and may not be sufficient for the generalisation of improved vocal function in habitual connected speech. As it is described, it is the ‘sound we make when we say yes’, ergo, it is cueing a habitual phonatory task, while cueing only subtle muscular or physiological improvements in phonation. The use of features that prime improved vocal function, including a semi-occluded vocal tract [29], voice onset at resting expiratory level [91], and cueing for a clear and effortless voice [19], may not be sufficient in this technique as gross changes in voice quality and increased activation of muscles not usually activated in habitual phonation (e.g., low larynx and cricothyroid activation) are not cued. These features are, however, repeated in SVQ in which increased muscle activation and re-posturing of the larynx is also cued.

The finding of improved voice quality measures after OPT (/m/) and SVQ (/ŋ/) were taught and practised as single sounds was unexpected, as these tasks are individual sounds designed to assist the patient to re-posture the larynx for more optimal phonation, which is acquired (or re-acquired) as a new voice motor skill. They were not trained in connected speech and were not habitual speech task targets, and as such were not expected to generalise to habitual speaking after having just acquired the task (and met the target in a single sound). Consideration of these two techniques as active ingredients is therefore warranted. It is important to note that the effect size was calculated with *n* = 24, a rather small sample size. Significant findings in the CSID of the CAPEV3 phrase may be due to the increased sensitivity of CSID as a measure of voice quality. Therefore, the findings on OPT need further investigation in future studies.

#### 4.2.2. Effects of SVQ

The significant effect of SVQ (as measured in auditory-perceptual ratings and the CPPS of CAPEV3) on the habitual speaking voice of patients after practising the SVQ in isolation was not predicted, given that the task itself was to acquire (not immediately generalise) the desired laryngeal adjustments of the technique and practise in preparation for the next exercise, which was task variation using SVQ. The improved voice quality in habitual speech was observed after the practising of an isolated sound suggests that the postural adjustments cued by the SVQ are possibly primary muscular movements of optimal phonation that could be considered active ingredients in themselves. Alternatively, the likely increased activation of both muscular and neural systems may also be implicated.

SVQ requires the production of a clear, quiet, and effortless ’ng’ sound, descending as if imitating a puppy whimper, to refine control of the optimal posture for phonation [30]. First described as ‘light’ registration by Vennard [92] and defined as “Falsetto break, expressive of grief” (p. 251) and “whine: Prolonged nasal or twangy sound, usually light in production, on descending portamento, expressing pain or disappointment” (p. 251), SVQ has subsequently been investigated as a voice quality mode named ‘cry’, compared to three other voice quality modes (speech, twang, and opera) [93]. Biomechanical and postural features observed in cry include low larynx position, increased space between the hyoid and thyroid, pharyngeal/supraglottic widening, increased aryepiglottic space, elongation of vocal folds, arytenoids not being tightly adducted, gentle and brief vocal fold closure, and possible increased activity of the cricothyroid and posterior crico-arytenoid [93]. Nearly all of these muscular parameters have been implicated in MTVD, including a raised larynx position, narrow supraglottic region, hyperadduction of the true vocal folds [94], and decreased hyoid/thyroid ‘visor’ [95].

This physiological description of SVQ suggests that all three biomechanical dimensions of the larynx are manipulated concurrently (medio-laterally, anterio-posteriorly, and inferior-superior) to correct the common biomechanical features of MTVD, with the added element of possibly activating the secondary neurological vocal pathway responsible for emotional vocalisation, as described by Simonyan [96]. Auditory–perceptual and kinaesthetic training is provided and encouraged in practice to link perception and production links in the vocal system [97,98]. More efficient learning and re-organisation of motor movements has been demonstrated in other domains to require maximal tolerable task complexity [99,100] and ability to recognise the target so that an internal reference of correctness is established for effective practice [41]. SVQ is a complex muscular task, the sound of which does not resemble habitual phonation (often a criticism of patients) but is perceptually recognizable and distinct from habitual phonation. This may promote increased recognition of the target (clear and effortless voicing) more readily than voicing in habitual conversation speech, in which the suboptimal phonation automatically occurs, assisting in generalisation.

#### 4.2.3. Effects of SVQ Task Variation

Task variation of carrier phrases and sirening in SVQ was used in this treatment to generalise the features of clear voice quality and the perceptions of effortless phonation to contexts other than/ŋ/. Results confirmed, as hypothesised, that task variation was effective in improving habitual voice quality across auditory–perceptual and acoustic analysis outcome measures. This was hypothesised based on a large body of previous research in voice therapy and motor learning, as task variation is considered essential in the learning, generalisation, and maintenance of all motor skills [101,102], despite the use of SVQ in the task. Task variation using connected speech tasks such as phrases and conversational speech is common across voice therapy approaches [25]. The explicit vocal target, use of connected speech contexts with a communicative intent, and practise regimes of SVQ are similar to other voice therapies, e.g., CTT (clear speech), but the physiological mechanism by which it is achieved is extremely different. This suggests that the mechanism of action [18] as a concept could be expanded to include the physiological description of movement as well as the acquisition and learning processes.

#### 4.2.4. Effects of Negative Practice

The NP component of SVT was highly effective across outcome measures based on results from both the mixed model and the ANOVA analysis of effect size. This was observed in auditory–perceptual outcomes in patients with primary and secondary MTVD, and across the whole cohort in acoustic measures. Negative practice (also called old way/new way) is thought to be a form of proactive interference that promotes forgetting of the old movement [103] and is commonly used in SLP and voice therapy [25,104,105]. The plateau in outcome from SVQ variants and NP may be explained by the function of NP to maintain the improvements resulting from SVQ variants, which may have resulted in a reduction in performance in the short term in some cases. As NP reintroduces the ‘old’ pre-treatment movement pattern, it is also possible that the performance parameters of the ‘new way’ are temporarily shifted until a clear differentiation between the generalised motor program for the two voice modes are well established. It is therefore conceivable that one session of NP with subsequent practise may have temporarily destabilised consistent access to the improved technique, resulting in temporary reduction in voice quality. As NP is designed to assist with generalisation and maintenance of a newly acquired skill and to extinguish access to the old suboptimal movement, an improvement in voice quality may not occur but rather a stabilisation of improvement may be more likely, as was observed in this study. Analysis of subsequent sessions is required to evaluate if habitual voice quality returned to post-SVQ levels and was retained in the long term.

### 4.3. Effect of Diagnosis and Service Delivery

Diagnosis of primary or secondary MTVD had a significant effect on auditory–perceptual voice ratings of strain only and was consistent over the four stages of the treatment. The clinical population in this study was typical of other MTVD cohorts reported in the literature, with retention rates also similar to other studies in which therapy was provided at no charge. There is significant evidence across RCTs and clinical studies that the retention of clients in voice therapy is generally poor [106]. Although the consequence of this is undocumented, high attrition runs the risk of ineffective treatment outcomes if the session dosage for the therapeutic effect is insufficient. In this study, positive therapeutic effects were observed across multiple voice outcomes within one to two sessions of 60-min durations with minimum durations of 1–2 weeks. If positive effects can be measured and demonstrated to patients within these short time frames, it is hoped that this would reduce attrition and increase compliance with further therapy recommendations.

Researchers have speculated that clinicians can have a therapeutic effect independent of the treatment type [107]. This is the first study to evaluate whether therapy delivered by multiple clinicians has a significant effect on voice outcomes. In this study, neither clinician, length of time, nor number of sessions had a significant effect on efficacy. This suggests that the active ingredients and overall efficacy of SVT are independent of the clinician, number of sessions, and length of treatment.

### 4.4. Comparison with Other Voice Therapy Outcomes Research

Comparison of effects found in this study with other treatments for patients with MTVD are difficult to make given the large range of outcome measures and different statistical analyses used across studies [11]. Numerous RCTs and prospective studies report a reduction in auditory–perceptual rating scores and improved acoustic analysis measures of voice quality including HNR, CPP, and CSID. Two retrospective cohort studies were found investigating the efficacy of VFE on patients with age-related dysphonia [108,109], only one of which documented the therapeutic outcome on voice quality auditory–perceptual and acoustic measures [109]. Small to medium effect sizes using Hedge’s ‘h’ were reported across a number of prospective and retrospective studies for improvements in voice quality outcome measures (shimmer and jitter only) after therapy, utilising VFE in patients with voice disorders [110]. Only one voice therapy treatment study reported using a mixed-model statistical analysis to measure voice outcomes across multiple time points in a prospective study of CTT with patients with mild MTVD who were stimulable for CTT [106]. This study reported significant effects of 4 weekly sessions, conducted no more than 10 days apart, using CTT. Five outcome measures were comparable with our study, including auditory–perceptual ratings using the CAPE-V, mean F0, CPP and CSID of a prolonged vowel, and CSID on the third CAPE-V phrase (amongst other outcome measures). Increases in mean F0 and reductions in CAPE-V ratings of the six CAPE-V phrases were reported. Effect sizes for significant effects were not reported, however. Baseline measures of the cohort in the CTT study were similar for mean F0 and the CPP vowel; however the CSID of the vowel and the third CAPE-V phrase were lower in our study. Significant improvements in habitual phonation as measured by acoustic voice analyses (CPP and CSID) were measured during and 1 week after the CTT therapy, but was not retained at 3 months. While the average number of sessions, average time between sessions, and practise recommendations were similar between the two studies, the retrospective nature of our study and the use of multiple clinicians meant that there was less control of the treatment variables, as it occurs in real-life clinical contexts.

We used both CPP (measured from ADSV) and CPPS (measured from Praat) to ensure that researchers can compare their data with the present study depending upon which software is available to them. Although ADSV is a commercial specialized software for clinical application, it is not accessible/available to many users, especially the non-clinicians, while Praat is a freeware. The discrepancy between the CPP and CPPS results for the CAPEV3 task (Table 7) probably resulted from the slight differences in the algorithms between these two programs rather than from the effects of the data distribution. CPPS showed more significant effects of treatment as the smoothing is believed to improve the cepstral estimation accuracy [73]; therefore, it would be more likely to detect finer changes in the voices given the mild dysphonic severity of the study cohort.

## 5. Conclusions

SVT was effective in reducing the signs and symptoms of mild MTVD in a typical treatment-seeking cohort, as measured by auditory–perceptual and acoustic voice outcomes. Three out of four individual components of the therapy program demonstrated statistically significant positive therapeutic effects, independent of the session number, duration of therapy, and clinician. This provides preliminary evidence that the SVQ technique and both the SVQ task variation and NP can be considered as active ingredients in the treatment of patients with MTVD.

## Figures and Tables

**Table 1:** Name and brief description of each of the first four Sob Voice Therapy components.

Component	Description
Optimal phonation task (OPT)	The patient is instructed to breathe in and out, then produce a clear, effortless, and quiet/m/using the sound we make when we mean ‘yes’. Instructions are given to prime the vocal system for low effort and low impact phonation including a gradual start (simultaneous onset). Focus is on ensuring the sound has communicative intent and is not produced as in singing. The patient is cued to notice how the sound feels and sounds. Explicit instruction is provided if whole-task modelling and imitation is insufficient for the patient to acquire the task. Home practise is recommended, ten repetitions/hour for 10 h during the day.
Sob voice quality (SVQ)	The patient is instructed to produce a clear, quiet, and effortless/ŋ/using a gradual start to the sound and a sad, mournful expression (similar to a puppy whimper). Explicit instruction is provided to cue increased accessory muscle activation if whole-task modelling and imitation is insufficient for the patient to acquire the task. The patient is cued to notice how their voice feels and sounds. Home practise is recommended from six to eight repetitions/hour for 10 h during the day.
SVQ task variation (SVQ variants)	The patient is instructed to produce all voice carrier phrases beginning with a momentary/ŋ/using SVQ. Phrases begin with all voiced sounds and then phrases with voiceless sounds are introduced. The patient is taught to produce a siren using a clear, quiet, and effortless/ŋ/using SVQ, slowly, smoothly, evenly, and effortlessly sliding the pitch up and down in the middle of their comfortable vocal range. Siren extensions that gradually increase and decrease pitch in the siren are also introduced. The patient is cued to notice how their voice feels and sounds. Home practise is recommended with six phrases/hour and two to three sirens/hour for 10 h during the day.
Negative practice (NP)	The patient is instructed to imitate the voice quality they presented with at assessment by listening to their initial voice recording. They are instructed to use this ‘old voice’ quality in carrier phrases used in SVQ task variation and then compare this with SVQ carrier phrases (still initiated with a momentary/ŋ/), which is the ‘new voice’. They are then asked to describe the differences between the two voice qualities with a focus on the sound and feeling of the voice. Home practise is recommended using three to four negative practice pairs (old way/new way) of SVQ phrases/hour for 10 h during the day.

NB: Practise recommendations are cumulative over the four components. Patients are instructed to randomise practise tasks in hourly practise sessions as different tasks are introduced.

**Table 2:** Number of sessions and days between each technique and process of the Sob Voice Therapy. Abbreviations: SD, standard deviation, and CI, confidence interval.

	OPT-SVQ *n* = 64		SVQ-SVQ Variants *n* = 43		SVQ Variant-NP *n* = 33		NP Post-NP *n* = 24		Total OPT Post-NP	
	Sessions	Days	Sessions	Days	Sessions	Days	Sessions	Days	Sessions	Days
Mean (SD)	1.3 (0.6)	28.5 (27.6)	1.5 (0.9)	38.3 (48.2)	2.5 (2.3)	37.3 (27.0)	1.2 (0.5)	24.0 (17.0)	4.0 (3.0)	83.1 (59.2)
95% CI	1.2–1.5	21.5–35.5	1.2–1.8	24.0–52.6	1.6–3.3	27.0–47.6	1.0–1.4	16.7–31.4	3.2–4.8	68.1–98.1
Min–max	1.0–4.0	4.0–173.0	1.0–5.0	6.0–248.0	1.0–11.0	7.0–105.0	1.0–3.0	7.0–84.0	1.0–15.0	7.0–283.0
Median	1.0	21.0	1.0	23.5	2.0	26.0	1.0	23.0	3.0	72.5

**Table 3:** Intra-rater reliability of the perceptual analysis (*p* < 0.001 for all measures).

Rater	Types of Measures	ICC			
		Severity	Roughness	Breathiness	Strain
Rater 1	Single measures	0.854	0.869	0.738	0.862
	Average measures	0.921	0.930	0.849	0.926
Rater 2	Single measures	0.977	0.889	0.948	0.896
	Average measures	0.988	0.941	0.974	0.945
Rater 3	Single measures	0.822	0.812	0.810	0.829
	Average measures	0.903	0.896	0.895	0.906

**Table 4:** Inter-rater reliability of the perceptual analysis.

Voice Measure	ICC Measures	ICC	95% CI	*p*
Overall severity	Single measures	0.703	0.547–0.824	0.000
	Average measures	0.876	0.783–0.933	0.000
Roughness	Single measures	0.696	0.537–0.819	0.000
	Average measures	0.873	0.777–0.932	0.000
Breathiness	Single measures	0.659	0.490–0.795	0.000
	Average measures	0.853	0.743–0.921	0.000
Strain	Single measures	0.691	0.531–0.816	0.000
	Average measures	0.870	0.772–0.930	0.000

**Table 5:** Fundamental frequency data (Hz) of the cohort at baseline (*n* = 68).

		Norms	Mean (SD)	95% CI	Min–Max
F0 of CAPEV3	Male	108.94 [83]	137.5 (31.2)	111.5–163.6	107.3–199.7
	Female	235.07 [83]	189.2 (17.7)	184.5–193.8	148.1–232.9
F0 of RP	Male	84–178 [40]	140.6 (43.3)	104.4–176.7	97.6–236.9
	Female	127–275 [40]	185.8 (14.7)	181.9–189.7	148.9–219.6
F0SD	Male	3.3 [84]	1.8 (1.0)	1.0–2.6	0.8–4.0
	Female	20–29y: 3.8 [85] 30–40y: 2.5 [86] 40–50y: 2.8 [86] 60–69y: 4.3 [85]	2.3 (1.4)	2.0–2.7	0.7–8.2

**Table 6:** Auditory–perceptual outcomes after four stages of Sob Voice Therapy. Partial η^2^ = 0.01, 0.1, and 0.25 indicate small, medium, and large effects, respectively. Abbreviation: MD, mean difference; (*), significance at *p* < 0.05.

Measures	Time Point	Mean (SD)	95% CI for Mean	MD	F	*p*	Partial η^2^
Overall severity	Baseline	26.8 (13.7)	20.2–33.4				
	OPT	25.8 (11.3)	20.4–31.3	1.0	0.376	0.546	0.018
	SVQ	20.0 (7.9)	16.2–23.8	6.8	12.001	0.003 *	0.387
	SVQ variant	20.9 (7.2)	17.4–24.4	5.9	12.381	0.002 *	0.360
	NP	21.6 (8.9)	17.3–25.9	5.2	11.312	0.003 *	0.340
Roughness	Baseline	24.1 (12.8)	17.9–30.3				
	OPT	22.8 (12.4)	16.8–28.8	1.3	0.865	0.363	0.041
	SVQ	19.5 (9.2)	15.1–23.9	4.6	7.069	0.016 *	0.271
	SVQ variant	18.8 (8.8)	14.5–23.0	5.3	12.289	0.002 *	0.358
	NP	19.4 (9.7)	14.7–24.1	4.7	10.471	0.004 *	0.322
Breathiness	Baseline	23.4 (13.8)	16.8–30.1				
	OPT	23.6 (11.1)	18.5–28.6	0.2	0.007	0.936	0.001
	SVQ	18.6 (8.2)	14.6–22.5	4.9	6.859	0.017 *	0.265
	SVQ variant	18.9 (5.9)	16.0–21.7	4.6	6.375	0.019 *	0.225
	NP	19.8 (8.1)	15.9–23.7	3.6	5.444	0.029 *	0.198
Strain	Baseline	18.4 (11.6)	12.8–23.9				
	OPT	16.8 (9.5)	12.2–21.3	1.6	1.526	0.231	0.071
	SVQ	17.0 (6.6)	13.8–20.2	1.3	1.085	0.311	0.054
	SVQ variant	12.3 (7.0)	8.9–15.7	6.1	17.713	0.001 *	0.446
	NP	13.6 (9.2)	9.2–18.0	4.8	9.409	0.006 *	0.300

**Table 7:** Acoustic outcomes after four stages of Sob Voice Therapy. Partial η^2^ = 0.01, 0.1, and 0.25 indicate small, medium, and large effects, respectively. Abbreviations: MD, mean difference and NA, not available; (*), significance at *p* < 0.05.

Measure	Normative Cut-Off	Time Point	Mean (SD)	95% CI	MD	F	*p*	Partial η^2^
HNR	20 dB [51]	Baseline	23.6 (5.4)	21.1–26.1				
		OPT	23.5 (6.1)	20.7–26.4	0.1	0.001	0.976	0.000
		SVQ	24.7 (4.9)	22.4–270.0	1.1	1.203	0.286	0.060
		SVQ variant	25.3 (4.7)	23.1–27.5	1.7	2.153	0.157	0.093
		NP	25.6 (4.2)	23.6–27.6	20.0	2.574	0.124	0.114
CPP of CAPEV3	>7.8 dB [87]	Baseline	7.1 (1.3)	6.5–7.7				
		OPT	7.2 (1.5)	6.5–80.0	0.2	1.147	0.296	0.052
		SVQ	7.4 (1.7)	6.6–8.1	0.3	0.995	0.331	0.050
		SVQ variant	7.4 (1.8)	6.6–8.3	0.4	1.563	0.224	0.064
		NP	7.7 (1.7)	6.9–8.5	0.6	50.098	0.034 *	0.188
CPPS of CAPEV3	NA	Baseline	10.6 (1.6)	9.9–11.4				
		OPT	11.2 (2.4)	10.2–12.3	0.6	1.752	0.200	0.077
		SVQ	11.6 (2.1)	10.6–12.6	10.0	6.208	0.022 *	0.237
		SVQ variant	120.0 (20.0)	11.1–12.9	1.4	10.587	0.003 *	0.315
		NP	12.1 (1.8)	11.3–12.9	1.5	17.629	0.001 *	0.445
CPP RP23	6.6 dB [87]	Baseline	5.6 (0.9)	5.1–60.0				
		OPT	5.5 (0.8)	5.1–5.9	0.1	1.126	0.292	0.017
		SVQ	5.8 (1.1)	5.3–6.4	0.2	0.806	0.375	0.019
		SVQ variant	5.9 (10.0)	5.3–6.4	0.3	50.009	0.032 *	0.135
		NP	60.0 (10.0)	5.5–6.5	0.4	3.577	0.071	0.135
CSID of CAPEV3	NA	Baseline	−130.0 (15.5)	(−20.2)–(−5.7)				
		OPT	−7.3 (17.3)	−15.4–0.9	5.7	50.096	0.035 *	0.195
		SVQ	−9.1 (14.6)	(−15.9)–(−2.2)	3.9	1.490	0.237	0.073
		SVQ variant	−8.7 (16.2)	(−16.2)–(−1.1)	4.3	2.416	0.134	0.095
		NP	−11.5 (16.4)	(−19.2)–(−3.8)	1.5	1.157	0.294	0.050
CSID of RP23	<24.27 [57]	Baseline	12.5 (12.3)	6.9–18.1				
		OPT	15.3 (13.9)	90.0–21.7	2.8	1.339	0.260	0.057
		SVQ	11.2 (14.7)	4.6–17.9	1.3	0.247	0.624	0.012
		SVQ variant	9.8 (14.4)	3.3–16.4	2.7	1.382	0.252	0.057
		NP	8.9 (13.5)	2.7–150.0	3.7	4.396	0.047 *	0.160

## Data Availability

Data supporting the reported results are retained by the University of Sydney in a deidentified form and is confidential under the conditions of the Human Research Ethics Committee of the University of Sydney approval.

## Abbreviations

MD, mean difference; CAPEV3, the third CAPEV phrase; RP, Rainbow Passage; RP23, second and third sentences of the Rainbow Passage; CPP, cepstral peak prominence; CPPS, cepstral peak prominence smoothed; and CSID, Cepstral/Spectral Index of Dysphonia.

## Appendix A. Settings for Acoustic Measurements

### Appendix A.1. Settings for the Fundamental Frequency Measurement in Praat

F0 range = 75–500 Hz, cross-correlation method, maximum number of candidates = 15, silence threshold = 0.03, voicing threshold = 0.45, octave cost = 0.01, octave-jump cost = 0.35, and voice/unvoiced cost = 0.14. The check box of “very accurate” was checked.

### Appendix A.2. Settings for the CPP Measurement in the Analysis of Dysphonia in Speech and Voice (ADSV)

Resampling rate = 25 kHz, spectral window size (pts) = 1024, maximum frequency for regression line calculation = 10,000, frame overlap = 75%, cepstral time-averaging (frames) = 7, CPP threshold (dB) = 0, and cepstral peak extraction range minimum–maximum = 60–300 Hz. Low/high spectral ratio cut-off = 4000 Hz.

### Appendix A.3. Settings for the CPPS Measurement in Praat

Pitch floor (Hz) = 60, time steps (s) = 0.002, maximum frequency (Hz) = 5000, pre-emphasis from (Hz) = 50, time-averaging window (s) = 0.01, quefrency-averaging window (s) = 0.001, peak search pitch range (Hz) = 60–330, tolerance (0–1) = 0.05, interpolation = parabolic, subtract tilt before smoothing = no, tilt line quefrency range (s) = 0.001–0.0 (=end), line type = straight, and fit method = robust.

## Appendix B

**Table A1:** Vocal load (missing data: *n* = 6).

Vocal Load	Frequency	Percent
Below 2 h	4	6.5
2–4 h	9	14.5
4–6 h	16	25.8
Above 6 h	33	53.2
Total	62	100.0

**Table A2:** History of voice-related comorbidities.

Comorbidities		Frequency	Percent
Reflux	No	37	54.4
	Yes	31	45.6
Sinusitis	No	52	76.5
	Yes	16	23.5
Asthma	No	57	83.8
	Yes	11	16.2
Cough	No	33	48.5
	Yes	35	51.5
Stress (missing data *n* = 4)	No	22	34.4
	Yes	42	65.6

**Table A3:** Lifestyle information.

Factors		Frequency	Percent
Caffein (missing data *n* = 2)	No	7	10.6
	Yes	59	89.4
Alcohol (missing data *n* = 1)	No	14	20.9
	Yes	53	79.1
Smoking (missing data *n* = 1)	No	65	97.0
	Yes	2	3.0

## Appendix C

**Table A4:** Changes in the acoustic outcomes before and after the four stages of Sob Voice Therapy. Partial η^2^ = 0.01, 0.1, and 0.25 indicate small, medium, and large effects, respectively.

Measure	Normative Cut-Off	Time Point	Mean (SD)	95% CI	MD	F	*p*	Partial η^2^
CPP vowel	11.74 dB [87]	Baseline	11.4 (2.6)	10.0–12.7				
		OPT	9.7 (2.6)	8.4–11.1	1.7	0.722	0.399	0.011
		SVQ	10.3 (2.3)	9.0–11.5	1.1	0.096	0.759	0.002
		SVQ variant	10.4 (2.1)	9.2–11.4	1.0	0.514	0.479	0.017
		Post NP	10.9 (2.1)	9.7–12.0	0.5	0.599	0.448	0.029
CPPS vowel	14.45 dB [111]	Baseline	14.5 (3.2)	12.8–16.1				
		OPT	13.3 (3.9)	11.4–15.3	1.2	0.658	0.420	0.010
		SVQ	13.4 (2.6)	12.1–14.7	1.1	1.215	0.277	0.029
		SVQ variant	13.6 (1.8)	12.6–14.5	0.9	0.197	0.661	0.007
		NP	13.7 (1.8)	12.8–14.6	0.8	1.054	0.317	0.050
CPPS RP23	9.33 dB [111]	Baseline	8.4 (1.0)	7.9–8.9				
		OPT	8.4 (1.0)	8.0–8.9	0.0	0.497	0.483	0.007
		SVQ	8.7 (1.3)	8.1–9.3	0.3	0.647	0.426	0.015
		SVQ variant	8.6 (1.1)	7.7–8.8	0.2	2.622	0.115	0.076
		NP	8.8 (1.2)	8.2–9.4	0.4	3.108	0.091	0.119
CSID vowel	NA	Baseline	3.8 (16.6)	−5.1–12.6				
		OPT	12.7 (17.3)	3.5–22.0	8.9	0.287	0.594	0.004
		SVQ	6.1 (11.5)	0.0–12.2	2.3	0.094	0.760	0.002
		SVQ variant	6.2 (12.8)	−0.6–13.0	2.4	0.935	0.341	0.030
		NP	3.4 (11.1)	−2.6–9.3	0.4	0.001	0.973	0.000
Intensity vowel	73.42 dB [76]	Baseline	57.0 (7.0)	53.4–60.6				
		OPT	56.4 (7.5)	52.6–60.3	0.6	1.147	0.288	0.018
		SVQ	57.9 (6.4)	54.6–61.2	0.9	0.253	0.618	0.006
		SVQ variant	58.0 (6.4)	54.7–61.3	1.0	0.118	0.734	0.004
		NP	56.8 (6.9)	53.3–60.4	0.2	0.055	0.817	0.003
Intensity CAPEV3	NA	Baseline	56.6 (5.3)	53.9–59.2				
		OPT	57.0 (6.8)	53.6–60.4	0.4	0.474	0.494	0.007
		SVQ	56.8 (6.0)	53.8–59.8	0.2	0.837	0.366	0.020
		SVQ variant	56.8 (5.6)	54.1–59.6	0.2	0.034	0.854	0.001
		NP	54.9 (6.3)	51.8–58.0	1.7	1.155	0.294	0.050
Intensity of RP	68.37 dB [112]	Baseline	47.9 (6.7)	44.7–51.1				
		OPT	49.3 (6.0)	46.4–52.2	1.4	0.369	0.545	0.006
		SVQ	49.5 (5.4)	46.9–52.1	1.6	0.035	0.852	0.001
		SVQ variant	49.0 (6.5)	45.9–52.1	1.1	0.036	0.851	0.001
		NP	47.8 (7.8)	44.0–51.6	0.1	0.006	0.938	0.000
